# How to Optimize Goal-Directed Medical Therapy (GDMT) in Patients with Heart Failure

**DOI:** 10.1007/s11886-024-02101-x

**Published:** 2024-08-02

**Authors:** Emily Newman, Chukwuemezie Kamanu, Gregory Gibson, Yevgeniy Brailovsky

**Affiliations:** 1 Division of Cardiology, Thomas Jefferson University Hospital, 833 Chestnut Street, Suite 630, Philadelphia, PA 19107 USA; 2 Department of Medicine, Thomas Jefferson University Hospital, 833 Chestnut Street, Suite 630, Philadelphia, PA 19107 USA

**Keywords:** Heart failure reduced ejection fraction, Systolic heart failure, Medications, Guidelines, Guideline directed Medical therapy

## Abstract

**Purpose of Review:**

Heart failure is a clinical syndrome with signs and symptoms from underlying cardiac abnormality and evidence of pulmonary or systemic congestion on laboratory testing or other objective findings (Bozkurt et al. in Eur J Heart Fail 23:352–380, 2021). Heart failure with reduced ejection fraction (HFrEF), when heart failure is due to underlying reduction in ejection fraction to $\le$ 40. The goal of this review is to briefly describe the mechanisms and benefits of the various pharmacological interventions described in the 2022 AHA/ACC/HFSA Guidelines focusing on Stage C: Symptomatic Heart Failure HFrEF, while providing basic guidance on safe use of these medications.

**Recent Findings:**

Use of medications from each class as recommended in the 2022 Guidelines can provide significant morbidity and mortality benefits for our patients.

**Summary:**

Despite advances in therapeutics for patients with HFrEF, patients are frequently under treated and more research is needed to help optimize management of these complicated patients.

## Introduction

**Fig. 1:**
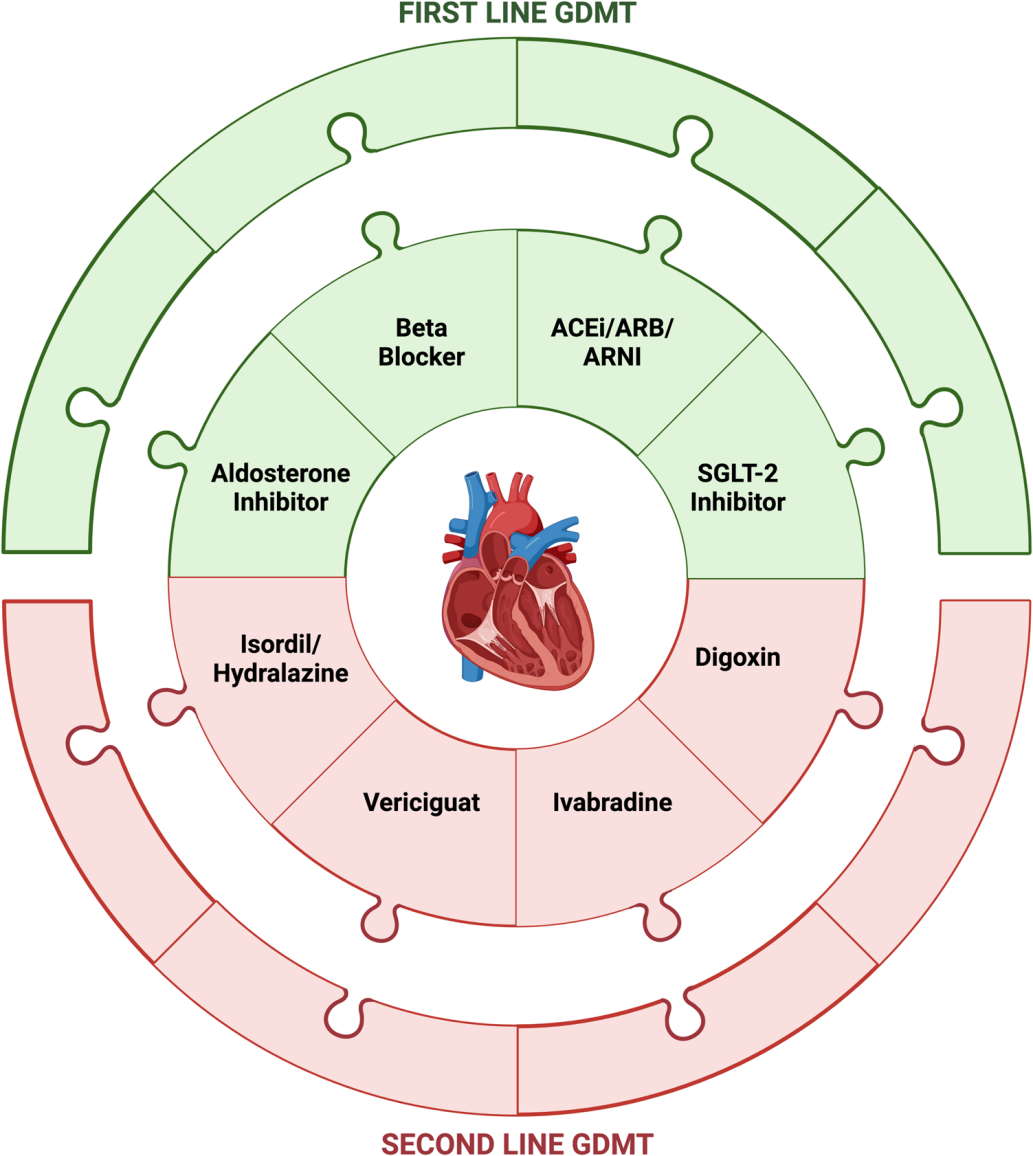
**Central Illustration**: First line Guideline Directed Medical Therapy (GDMT) having class I recommendations in the 2022 AHA/ACC/HFSA guidelines for Heart Failure with Reduce Ejection Fraction (HFrEF) as well as second line therapies with class IIa and IIb recommendations. Angiotensin converting enzyme inhibitor (ACE), angiotensin receptor blockers (ARB), angiotensin receptor neprolysin inhibitor (ARNI) and sodium-glucose cotransportor-2 (SGLT-2). The figure was created using BioRender.com

Heart failure is a clinical syndrome with signs and symptoms from underlying cardiac abnormality and evidence of pulmonary or systemic congestion on laboratory testing or other objective findings [1]. Heart failure with reduced ejection fraction (HFrEF), when heart failure is due to underlying reduction in ejection fraction to $\le$ 40%, is a growing issue in the United States and globally as the population ages and comorbid risk factors increase. There is significant morbidity and health care expenditure with this diagnosis as patients are frequently hospitalized and mortality is high. Initially, HFrEF was treated with digoxin and diuretics but as the understanding of the pathophysiology of heart failure has evolved, more targeted treatments which improve morbidity and mortality in patients with HFrEF have been developed. These therapies included beta blockers (BB), mineralocorticoid receptor antagonists (MRA), angiotensin converting enzyme inhibitors (ACEi), angiotensin receptor blockers (ARB), angiotensin receptor neprolysin inhibitor (ARNI) and sodium-glucose cotransportor-2 (SGLT-2) inhibitors. These medications are commonly referred to as the “four pillars” of HFrEF management, or as Guideline-Directed Medical Therapy (GDMT). Sometimes, these are mistakenly referred to as “Goal-Directed Medical Therapy,” which leads to opposition in some who insist that all therapies should be driven by a goal. But perhaps “Goal-Directed Medical Therapy” is a better term. As we move towards individualized patient care, the medicines we prescribe should be aligned with the goals of our patients and, for some of our sickest patients, that goal may be decreased morbidity even if that comes at the expense of reduced survival. Regardless of the terminology, the goal of this review is to briefly describe the mechanisms and benefits of the various pharmacological interventions described in the 2022 AHA/ACC/HFSA Guidelines focusing on Stage C: Symptomatic Heart Failure HFrEF, while providing basic guidance on safe use of these medications (Fig. 1 Central Illustration).

## *Beta* Blocker

Heart failure causes hyperactivation of the sympathetic nervous system (SNS) as well as the renin–angiotensin–aldosterone system (RAAS), which perpetuates cardiac dysfunction and contributes to adverse cardiac remodeling [2]. Beta blockers reverse remodeling and have been the mainstay for management of HFrEF since the 1990s with several landmark trials published, including CIBIS and CIBIS II for bisoprolol, MERIT-HF for metoprolol, and COPERNICUS for carvedilol [3–6]. These trials demonstrated a mortality benefit of 23–34% with the use of beta blockers in patients with HFrEF. The 2022 AHA/ACC/HFSA guidelines have a Class 1A recommendation for the use of beta blockers (metoprolol succinate, carvedilol, or bisoprolol) in patients with HFrEF [7]. The benefit of beta blockers extends beyond just reduction in HR and should be increased to target dose or maximally tolerated dose [8]. Target doses are carvedilol 25-50mg twice daily, bisoprolol 10mg daily, and metoprolol succinate 200mg daily [9]. If clinical status permits, doses can be increased every 1–2 days for inpatients or every two weeks for outpatients [10, 11].

Beta blocker initiation is best when patients are euvolemic [10] and studies suggest that blocker discontinuation in patients with acute decompensated heart failure worsens mortality and rehospitalization rates. Beta blockers should be continued if patient confirms compliance and in the absence of hypotension, shock, or severe pulmonary edema [12]. Similarly, dose titration to goal dose is limited by bradycardia and hypotension in some patients [2].

There have been several studies comparing the approved beta blockers. The COMET trial, published in 2003, was a randomized double-blinded trial which demonstrated that carvedilol had a superior effect on mortality when compared to metoprolol [13]. However, this trial compared the tartrate formulation of metoprolol, rather than succinate, and at lower equivalent doses than the MERIT-HF trial. Subsequent retrospective trials have provided differing findings, and more data is needed to determine if any particular beta blocker has superior outcomes [14, 15].

The selective β1 receptor antagonists (bisoprolol and metoprolol) are preferred in patients with chronic obstructive pulmonary disease (COPD) or asthma, while the nonselective carvedilol is preferred in patients with arterial hypertension [2]. Beta blockers in patients with diabetes mellitus can potentially impair insulin release or mask the catecholamine-mediated symptoms of hypoglycemia, but studies have shown use of carvedilol and bisoprolol in this population [2]. Table 1 lists starting and target doses for the approved beta blockers as well as dose adjustments required for renal dysfunction. Carvedilol is contraindicated by the manufacturer in the setting of severe hepatic dysfunction but is still used by experts for a variety of conditions. Bisoprolol and metoprolol are not contraindicated in hepatic dysfunction but may require slower dose titration to ensure clinical safety.

**Table 1:** Starting and target doses for the approved beta blockers and dose adjustments required for renal dysfunction

Medication	Starting dose	Target dose
Carvedilol	3.125mg BID	25-50mg BID
- Renal dysfunction	No adjustment	No adjustment
Bisoprolol	1.25mg daily	10mg daily
- Renal dysfunction CrCl < 20 ml/min/1.73m2	No dose adjustment, may need slower titration	No dose adjustment, may need slower titration
Metoprolol Succinate	12-25mg daily	200mg daily
- Renal dysfunction	No dose adjustment	No dose adjustment

## ACE Inhibitor/Angiotensin Receptor Blocker/Angiotensin Receptor-Neprilysin Inhibitor

The renin-aldosterone-angiotensinogen (RAAS) system involves angiotensinogen, which is converted to angiotensin I by renin. Angiotensin I is converted to angiotensin II by angiotensin-converting enzyme (ACE). Angiotensin II is the active molecule which binds to the AT1 receptor, leading to downstream effects such as vasoconstriction, aldosterone secretion, and sodium retention. In the heart, AT1 activation leads to cellular hypertrophy that results in fibrosis, which ultimately contributes to ventricular remodeling. Aldosterone binds to the mineralocorticoid receptor, leading to sodium and water retention in the kidney with excretion of potassium to maintain blood pressure [16].

ACE inhibitors (ACEi) block the conversion from angiotensin I to angiotensin II and were first shown to improve mortality in patients with NYHA class IV HFrEF in 1987 in the CONSENSUS trial, with a 31% reduction in mortality at one year [17]. Subsequently in 1991, the SOLVD trial demonstrated that enalapril reduced mortality and hospitalizations for patients with HFrEF and NYHA class II-III symptoms [18]. Due to medication intolerance with ACEi, use of angiotensin receptor blockers (ARBs), which block the AT1 receptor, were performed. These studies demonstrated that ARBs were noninferior to ACEi and combinations of both could further reduce morbidity [19, 20]. The 2013 ACCF/AHA Guideline for the Management of Heart failure recommended routine use of ACEi to reduce mortality and morbidity in patients with HFrEF. ARB could be used in place of ACEi in intolerant patients and could be used in addition to ACEi in patients who could not tolerate MRA but the combination of ACEi, ARB, and MRA is potentially harmful when all three are used simultaenously [21].

Neprilysin degrades vasoactive peptides including natriuretic peptides and bradykinin. Inhibition of neprilysin helps to counteract the detrimental effects of neurohormonal overactivation in chronic heart failure that leads to vasoconstriction, sodium retention, and remodeling. With the PARADIGM-HF trial in 2014, a new agent, the angiotensin receptor-neprilysin inhibitor (ARNI)—sacubitril-valsartan, was found to be superior to enalapril with respect to mortality and hospitalizations in patients with HFrEF [16].

The 2022 AHA/ACC/HFSA guidelines provided a Class IA recommendation for ARNI in patients with HFrEF and NYHA class II-III symptoms to reduce morbidity and mortality. Use of an ACEi carries a Class IA recommendation for patients who are not able to tolerate ARNI due to side effects or cost. Similarly, use of ARB for patients who are unable to tolerate ACEi has a Class IA recommendation. In those patients with NYHA class II-II symptoms who are tolerating and ACEi or ARB, there is a Class IB-R recommendation to switch to ANRI to further reduce morbidity and mortality [7]. RAAS inhibition is tolerated when patients have volume overload and use is limited by hypotension, renal dysfunction, and electrolyte abnormalities. ACEi are often discontinued due to cough, and ARNI and ACEi are contraindicated in patients with angioedema. Renal function should be checked 1–2 weeks after dose initiation or modification [10]. Of note, use of ARNI within 36 h of last ACEi dose is contraindicated due to increased risk for adverse effects. ARNIs are not recommended patient with severe hepatic impairment (Child–Pugh Class C), and the remaining medications should be used with caution in patients with cirrhosis, but do not have specific dose alterations. Table 2 lists starting and target dosing for commonly used RAAS inhibitors included in the 2022 AHA/ACC/HFSA guidelines [7].

**Table 2:** Starting and target dosing for commonly used RAAS inhibitors and dose adjustments required for renal dysfunction

	Starting dose	Target Dose	Renal Function**
ARNI			
- Sacubitril valsartan*	24/26mg -49/51mg twice daily	97/103mg twice daily	< 30, half dose
ARB			
- Valsartan	20-40mg twice daily	160mg twice daily	No dose adjustment
- Candesartan	4-8mg daily	32 mg daily	CrCl < 30 mL/min: start 4mg daily, target 16mg daily
- Losartan	25-50mg daily	150mg daily	No dose adjustment
ACEi			
- captopril	6.25mg three times daily	50mg three times daily	CrCl 10–50 mL/min: dose every 12–24 h, max 50mg every 12 h CrCl < 10 mL/min: dose every 24 h, max 50mg every 24 h
- enalapril	2.5mg twice daily	10-20mg twice daily	CrCl 10-30mL/min: start 2.5mg daily in 1 or 2 divided doses, max dose 20mg/day CrCl < 10 ml/min: consider alternative, dose 1.25mg daily or 2.5mg every other day, max dose 10mg/day
- lisinopril	2.5-5mg daily	20-40mg daily	CrCl 10–30 ml/min: start 2.5mg daily CrCl < 10 mL/min: consider alternative agent
- Ramipril	1.25mg daily	10mg daily	GFR 15–30 ml/min/1.73m2: max dose 5mg daily GFR < 15 ml/min/1.73m2: consider alternative agent, max dose 5mg/day in 1–2 divided doses

^*^Sacubitril/valsartan is approved for NYHA class II-III HF while the remaining medications are recommended for class I-IV HFrEF

^**^ With decline in renal function, medications should be titrated slower with more frequent monitoring than patients with normal renal function

## Mineralocorticoid Receptor Antagonists

Aldosterone is part of the RAAS system and promotes sodium retention, sympathetic activation, and myocardial fibrosis. Mineralocorticoid receptor antagonists (MRAs) have been used to block these effects in patients with HFrEF and promote cardiac reverse remodeling. The RALES study demonstrated a mortality and morbidity benefit with the addition of spironolactone to patients with HFrEF on the background ACEi treatment [22]. Subsequently, the EPHESUS trial demonstrated mortality benefit with eplerenone, a selective aldosterone blocker, in patients with HFrEF after acute MI [23]. The EMPHASIS-HF trial further solidified the MRAs as a pillar of HFrEF therapy by demonstrating benefits in morbidity and mortality in patients with HFrEF and NYHA class II symptoms [24].

The 2022 AHA/ACC/HFSA guidelines give a Class IA recommendation for spironolactone or eplerenone to reduce morbidity and mortality in patients with NYHA class II-IV HFrEF. Renal dysfunction and hyperkalemia are significant side effects of MRAs, especially in patients also taking ACEi, ARB, or ARNI. Patients should have eGFR > 30 l/min/1.73m2 with potassium < 5 mEq/L at initiation and require close monitoring of renal function and electrolytes. The guidelines recommend checking laboratory markers or renal function at 1 week, 1 month, then every 6 months after initiation or dose adjustment of MRA. If potassium cannot be maintained < 5.5mEq/L while on an MRA the drug should be discontinued. Additionally, temporarily holding the MRA in settings of diarrhea significant enough to lead to dehydration or during pauses in loop diuretic therapy should be considered to prevent hyperkalemia. Eplerenone, as a more selective medication, may be better tolerated than spironolactone in patients at risk for gynecomastia or vaginal bleeding [7]. Neither medication requires dose adjustment for hepatic dysfunction. The initial dose for both medications is 25mg daily with GFR > 60ml/min/1.73m2. Target dose is 25-50mg daily for spironolactone and 50mg daily for eplerenone. Dose adjustment is required for kidney dysfunction and starting dose and target dose should be halved for GFR 30–60 ml/min/1.73m2 (start at 12.5mg daily, max 25mg daily). Spironolactone and eplerenone are contraindicated with GFR < 30 ml/min/1.73m2.

## Sodium-glucose Cotransporter-2 Inhibitors

Sodium-glucose cotransporter-2 inhibitors (SGLT-2) inhibitors were initially developed as medications to treat diabetes mellitus as they block proximal renal tubular glucose reabsorption, leading to increased glucosuria and lowering of hemoglobin A1c. Due to increased safety monitoring for antihyperglycemic medications, the SGLT-2 inhibitors were noted to have cardiovascular benefits which were further evaluated in several landmark trials. The DAPA-HF trial demonstrated improvement in mortality and heart failure hospitalizations for NYHA class II-IV patients with HFrEF when treated with dapagliflozin [25]. The EMPEROR-Reduced trial similarly demonstraed a reduction of the primary endpoint, a composite of cardiovascular death or first heart failure hospitalization for patients taking empagliflozin [26]. These benefits were independent of diabetes mellitus status in both trials [25, 26].

The mechanism for these benefits is not well understood. It is thought to be glucose-independent, and there are a range of proposed mechanisms including changes in cardiac oxygen demand, cardiorenal effects, or vascular changes which occur in response to this class of medications. The 2022 AHA/ACC/HFSA guidelines carry a Class IA recommendation for SGLT-2 inhibitors in patients with symptomatic chronic HFrEF to reduce hospitalizations and mortality regardless of type 2 diabetes mellitus status [7]. The DAPA-HF and EMPEROR-Reduced trials included patients with NYHA class II-IV HF [16, 26]. Both Dapagliflozin and Empagliflozin are dosed at 10mg daily. DAPA-HF did not include patients with eGFR < 25 ml/min/1.73m2 and EMPEROR-reduced did not include patients with eGFR < 20ml/min/1.73m2 [16, 26]. Patients require counseling on increased risk for genitourinary infections with these medications and they are contraindicated in patients with a history of Fournier’s gangrene. There is a risk of euglycemic diabetic ketoacidosis and patients with type 1 diabetes mellutis should not take SGLT-2 inhibitors [7]. While SGLT-2 inhibitors alone do not cause significant hypoglycemia, this risk can be increased in patients on concurrent insulin and sulfonylureas, and it may be beneficial for patients’ primary diabetes mellitus management team to be involved in these cases [10].

## Isosorbide/Hydralazine

The combination of venodilation and arteriolar dilation with hydralazine and isosorbide dinitrate were among the original medications that showed mortality benefit in patients with HFrEF. The Vasodilator Therapy in CHF, published in the 1980s, showed mortality benefit with the addition of hydralazine and isosorbide dinitrate when added to background digoxin and diuretics [27]. Subsequent studies did not demonstrate the same benefit when compared to ACEi therapy and hydralazine/isosorbide dinitrate, but further analysis suggested additional mortality benefit in Black patients [28]. The African American Heart Failure Trial (A-HeFT), published in 2004, was terminated early due to improved survival with the addition of hydralazine/isosorbide to patients who self-identify as Black and have NYHA Class III-IV HF with dilated cardiomyopathy and EF < 35% or EF < 45% with LVEDD > 2.9cm/m2 (or > 6.9cm) while on background therapy with beta blocks and ACE inhibitors [29]. The mechanism of benefit with this combination is thought to be related to alterations in preload and afterload as well as changes in the processing of nitric oxide. Indeed, differences in nitric oxide signaling has been suggested as an explanation for the differing effects depending on race [29].

The 2022 AHA/ACC/HFSA gGidelines provide a Class IA recommendation for the addition of hydralazine and isosorbide dinitrate to reduce morbidity and mortality in African American patients with NYHA class II-IV HFrEF otherwise on optimal medical therapy, while noting that this therapy has not been studied in combination with ARNI [7]. Drug dosing, similar to A-HeFT study is 37.5mg hydralazine and 20mg isosorbide dinitrate three times daily with doubling of dose to target 75mg hydralazine and 40mg isosorbide dinitrate three times daily. Alternatively split doses of 20-30mg isosorbide dinitrate and 25-50mg hydralazine 3–4 times daily with up titration to 120mg total isosorbide dinitrate and 300mg total hydralazine in divided doses is recommended if the combination pill is not prescribed. Dose adjustment is not required for renal or hepatic dysfunction. Hypotension is the primary limiting factor in medication use and isosorbide dinitrate is contraindicated in combination with PDE-5 inhibitors. The 2022 AHA/ACC/HFSA Guidelines also provide a Class 2B recommendation for the addition of hydralazine-isosorbide dinitrate in patients with current or previous symptomatic HFrEF who are unable to tolerate the other Class I-recommended agents. The benefit of this combination is uncertain, and the recommendation is based on the original Vasodilatory Therapy in CHF trial which demonstrated mortality benefit [7].

## Ivabradine

Elevated resting heart rate is a risk factor for mortality in patients with HFrEF. Heart rate > 70 bpm has been shown to have 34% increased risk of cardiovascular death and 53% increased risk of heart failure hospitalization. Ivabradine inhibits the *I*_f_ current in the sinoatrial node leading to reduction in heart rate without affecting cardiac contractility. The Ivabradine and outcomes in chronic heart failure (SHIFT) trial found that the addition of ivabradine to patients with EF < 35% and HR > 70 bpm despite maximum tolerated beta blocker dose led to a modest reduction in heart failure mortality and hospitalization at two years [30]. Based on this trial, the 2022 AHA/ACC/HFSA Guidelines carry a Class 2A recommendation for the addition of ivabradine to reduce heart failure hospitalizations and death in patients with NYHA class II-III stable chronic HFrEF with EF < 35% and on background optimal medical therapy, including maximumly tolerated beta blocker whose heart rate remains > 70 bpm while in sinus rhythm [7]. Ivabradine dosing in the SHIFT trial started at 5mg BID and was increased to target 7.5mg BID after two weeks if the HR remained > 60 bpm. Patients with resting HR 50–60 bpm were maintained at 5mg BID and the dose was reduced to 2.5mg BID for those with BP < 50bpm or with symptoms of bradycardia. It is worth noting that congenital heart disease and primary valvular heart failure patients were excluded from the SHIFT trial [30]. Ivabradine can cause vision changes, does not require dose adjustment in kidney disease and is contraindicated in severe liver dysfunction (Child–Pugh class C).

## Vericiguat

The nitric oxide-soluble guanylate cyclase pathway is another important pathway that is disrupted in heart failure. Normally, when stimulated, the intact endothelium can generate nitric oxide which diffuses into smooth muscle to trigger production of cyclic GMP from guanylate cyclase. Cyclic GMP stimulates smooth muscle relaxation and anti-proliferation in the vasculature. In cardiac tissue, it regulates contractility and relaxation making cyclic GMP critical signaling molecule in myocardial energics and performance [31]. In heart failure, this pathway is disrupted due to inflammation, endothelial dysfunction, and increased reactive oxygen species leading to reduced bioavailability of nitric oxide. Downstream, this leads to a relative deficiency in soluble guanylate cyclase and reduced generation of cyclic GMP.

Vericiguat is an oral agent that directly stimulates GMP production by binding to guanylate cyclase at a different site from nitric oxide while simultaneously sensitizing the enzyme to endogenous nitric oxide. The Vericiguat in Patients with Heart Failure and Reduce Ejection Fraction (VICTORIA) trial demonstrated reduction in the primary outcome, a composite of cardiovascular death and heart failure hospitalizations in patients with HFrEF. Patients in the VICTORIA trial had NYHA class II-IV symptoms, EF < 45%, elevated NT-proBNP, and a recent hospitalization for heart failure or required IV diuretics. The trial found a reduction in death or hospitalization after 3 months of treatment, primarily driven by a reduction in hospitalizations. There appeared to be no benefit for the subgroup with the highest NT-proBNP (> 5314 pg/ml) and SGLT-2 inhibitor use was low, so benefit in combination with vericiguat is less clear in this population. Patients on long-acting nitrates, PDE-5 inhibitors, IV inotropes, and LVADs were excluded from the trial [32].

The 2022 AHA/ACC/HFSA guidelines provides a Class 2B recommendation to consider vericiguat in select high risk or recently worsening HF patients on baseline medications to reduce hospitalizations and cardiovascular deaths [7]. Dosing of vericiguat in the VICTORIA trial started at 2.5mg daily with up titration every two weeks based on blood pressure. Doses were increased to target 10mg if SBP was > 100mmHg. Patients with SBP 90-100mmHg were maintained on current dose and those with SBP < 90 mmHg had doses decreased if on 5 mg or 10mg and doses held if on 2.5mg daily. Vericiguat can cause syncope, symptomatic hypotension, and anemia, and does not require dose adjustments for renal or hepatic dysfunction, although patients with GFR < 15 ml/min/1.73m2 were excluded from the trial [32].

## Digoxin

William Withering first described the use of digitalis for heart failure in 1785, making it the oldest cardiac medication still in use [33]. We now know that digoxin, a cardiac glycoside, acts as an inotrope by inhibiting myocardial Na–K ATPase causing increased intracardiac sodium concentrations, which leads to increased calcium influx and cardiac contractility. It is also a vagomimetic agent that promotes activation of the parasympathetic nervous system while inhibiting the sympathetic nervous system. It decreases the SA node automaticity and decreases conduction through the AV node. Digoxin decreases renin-angiotensin activation but may stimulate aldosterone release. Overall, these effects lead to increased cardiac output with decreased right and left sided pressures [34].

Data on use of digoxin with modern HFrEF regimens is limited. The Digitalis Investigator Group (DIG) evaluated the effect of digoxin in patients with EF < 45% on ACEi and diuretics and found a reduction in overall and heart failure hospitalizations but no change in mortality. In subgroup analysis, the benefits were greatest for those with EF < 25%, those with enlarged hearts, and those with NYHA class III and IV [35]. The 2022 AHA/ACC/HFSA Guidelines give a Class 2B recommendation for addition of digoxin to patients with Stage C HFrEF who remain symptomatic despite optimal background therapy, as it may reduce hospitalizations. The guidelines acknowledge limited data on the benefit for patients on current HF medications. Dose recommendations are to maintain low doses of 0.125 or 0.25mg daily for target serum levels of 0.5–0.9 ng/ml. Patients older than 70 years old, with renal dysfunction, or low lean body mass are recommended to start at lower doses of 0.125mg daily or every other day. Dosing nomograms based on ideal body weight and creatinine clearance are available [36]. Digoxin toxicity, which can cause fatal arrythmias, requires close monitoring of drug levels as well as electrolytes and kidney function, especially in the setting of active diuresis. Of note, digoxin discontinuation is associated with worse clinical outcomes, including one study which found an increased rate of hospitalizations starting 6 months after drug discontinuation and persisting the 4 years of the trial period. This study also found increased mortality in the 30 days – 1 year after digoxin discontinuation [37].

## Overall Medication Benefits and Utilization

Use of medications from four major drug classes: ARNI/ACEi/ARB, Beta blocker, MRA, and SGLT-2i is estimated to reduce mortality by 73% compared with no medications [7]. One trial estimated that a 70-year-old patient with HFrEF on ARNi, BB, MRA and SGLT-2 would gain five years of life compared to no therapy [38]. As described in the 2022 guidelines, ACEi/ARB/ARNI have a 16–17% relative risk reduction in mortality. Beta blockers, MRA and SGLT2i have 34%, 30% and 17% relative risk reduction. Hydralazine and isosorbide dinitrate has a 43% relative risk reduction in mortality [7]. Despite these benefits, patients frequently are not on optimal medical therapy. A registry study from 2018 (before SGLT-2 were recommended for HF) found that of more than 3,500 HFrEF patients, 27% were not prescribed an ACEi, ARB or ARNI, 33% were not on a beta blocker and 67% were not prescribed an MRA [39]. A study of prescription patterns for Medicare beneficiaries found that less than 40% of patients fill prescriptions for beta-blockers despite only 10% of patients having a potential contraindication of COPD, asthma, or syncope [40].

The guidelines note that every attempt should be made to reach target goals for the recommended medications and repeated attempts to increases doses can result in success. Dose titrations are described based on trial methods but should be tailored to individual patients while avoiding delays in reaching target dose [7]. The STRONG-HF trial was a randomized controlled trial that evaluated patients hospitalized for acute HF exacerbation (regardless of EF) to usual care vs the high intensity arm where patients were started on ACEi/ARB, beta blocker, MRA and up titrated to 50% target dose by discharge and to 100% target dose by 2 weeks of discharge. It found reduced symptoms and improved quality of life with reduced risk of death or readmission at 180 days in the treatment group [41]. Subsequent analysis found no difference in outcomes or adverse events after stratifying by EF [42]. There is some debate as to whether to initiate all four medication classes (Beta blocker, ACEi/ARB/ARNI, MRA, SGLT-2i) at once or have a more stepwise approach. The benefit of starting multiple agents at once is the ability to reach target doses quicker. But there are also several drawbacks to such a strategy. The specific approach likely varies from patient to patient and is dependent on several factors such as patient’s heart rate and blood pressure, renal function, potassium level, among others. It also matters what clinical setting the patient is in. It may be easier to initiate more drugs simultaneously on the in-patient setting, where we can carefully watch for adverse events and monitor vital signs and laboratory markers closely, as compared to outpatient setting where monitoring may be limited. Also chosen approach likely varies with the comfort level of a physician and patient. Finally, some providers choose to initiate therapies one at a time in case adverse events arise, they can be more easily attributed to a particular therapy, which can then be reduced or discontinued, instead to having to stop multiple agents at once.

## Conclusion

Heart failure with reduced ejection fraction is a common condition with high hospitalization and mortality rates [43]. Advances in medical management for HFrEF allow for reducing symptoms and decreasing mortality. Use of beta blockers, ARNI, MRA and SGLT-2 inhibitors at target dosing is estimated to reduce mortality by 73% or estimated to prolong the life of a 70-year-old by 5 years compared with no medications [7, 38]. Table 3 summarizes the landmark trials for the different medications as well as the number needed to treat for all-cause mortality at 12 and 36 months based on those trials. Despite these advances in heart failure care, significant numbers of patients do not receive these life changing treatments [44]. All patients with HFrEF should have every effort made to reach maximal doses of beta blockers, ARNI, MRA and SGLT-2 inhibitors. The other medications we described (ivabradine, isordil/hydralazine, digoxin, vericiguat) can be used as adjunct second line agents when symptoms persist despite GDMT or if first line agents are contraindicated. Medications can be initiated sequentially or simultaneously depending on the individual patients clinical status and should be quickly uptitrated [45]. Ultimately, further work is needed to improve utilization of guideline recommended heart failure treatments to help patients achieve the goal of a longer life with fewer symptoms.

**Table 3:** Heart Failure Landmark Trials [4–7, 16–20, 22, 24–26, 29]

Drug Class	Medication	Trial	Year published	RRR for all-cause mortality	NNT for mortality 12 months	NNT for mortality 36 months
Beta blockers				34%	28	9
	Bisoprolol	CIBIS II	1999			
	Metoprolol Succinate	MERIT- HF	2000			
	Carvedilol	COPERNICUS	2001			
ARNI				16%	80	27
	Sacubitril-valsartan	PARADIGM-HF	2014			
ACEi				17%	77	26
	Enalapril	CONSENSUS	1987			
	Enalapril	SOLVD	1991			
ARB				17%	77	26
	Valsartan	Valsartan HF Trial	2001			
	Candesartan	CHARM	2004			
MRA				30%	18	6
	Spironolactone	RALES	1999			
	Eplerenone	EMPHASIS -HF	2011			
SGLT-2				17%	63	22
	Dapagliflozin	DAPA-HF	2019			
	Empagliflozin	EMPEROR-Reduced	2020			
Hydralazine/ Isosorbide				43%	21	7
	Hydralazine/Isosorbide	A-HeFT	2004			

RRR-Relative Risk Reduction, NNT-Number Needed to Treat
